# Oral Administration of Heat-Inactivated *Lactobacillus plantarum* K37 Modulated Airway Hyperresponsiveness in Ovalbumin-Sensitized BALB/c Mice

**DOI:** 10.1371/journal.pone.0100105

**Published:** 2014-06-17

**Authors:** Yen-Wenn Liu, Tan-Wei Liao, Yu-Han Chen, Yi-Chin Chiang, Ying-Chieh Tsai

**Affiliations:** Institute of Biochemistry and Molecular Biology, National Yang-Ming University, Taipei, Taiwan, ROC; French National Centre for Scientific Research, France

## Abstract

This study aimed to investigate the anti-allergic effects of *Lactobacillus plantarum* K37 (K37) on airway hyperresponsiveness (AHR) and systemic allergic responses in ovalbumin (OVA)-sensitized and -challenged BALB/c mice. Heat-inactivated K37 (10^5^, 10^7^, and 10^9^ CFU/mouse, day) were orally administered to OVA-sensitized BALB/c mice to investigate their effects on AHR, immunoglobulin (Ig) and cytokine production. The results showed that K37 dose-dependently lowered the serum levels of IgE, OVA-specific IgE and OVA-specific IgG1, ameliorated AHR induced by methacholine and suppressed eosinophil infiltration in bronchoalveolar lavage fluid (BALF). The cytokine production in spleen cells culture and BALF showed that K37 drove the immune responses toward T-helper cell type 1 (Th1) responses, elevated levels of IL-2 and IFN-γ, and reduced of IL-4, IL-5 and IL-13. K37 also improved cell infiltration in lung sections. Our results demonstrated that oral administration of K37 alleviated effectively the allergic responses *in* *vivo*. Thus, K37 can be a good source material and a promising candidate for prophylactic and therapeutic treatments of allergic diseases, like asthma.

## Introduction

Allergic disorders, such as allergic rhinitis [1], atopic dermatitis [2], food allergies [3], and allergic asthma [4], not only affect the individual’s life quality but also become a medical burden on society. Allergies are related to the T helper cell type 2 (Th2) responses which can be characterized by the production of cytokines, interleukin (IL)-4, -5, -13, the production of total immunoglobulin (Ig) E and antigen-specific Igs (i.e. IgE, IgG1 and IgG2) and the accumulation of eosinophils [5]. Among the Th2 cytokines, IL-4 and IL-13 are investigated the therapeutic intervention in asthma and other Th2-associated diseases. As for IL-13, it was reported to directly enhance mucus hypersection and airway hyperresponsiveness (AHR) [6]. Moreover, IL-5 is known to be important to the differentiation, maturation, and recruitment of eosinophils [7]. However, Th2 responses can be suppressed by T helper cell type 1 (Th1) cells which secreted interferon (IFN)-γ, IgG2a, IL-2, and IL-3 [8]. Therefore, to regulate the immune responses by suppressing Th2-responses while enhancing Th1-responses is expected to be helpful in the treatment of allergy and other Th2-dominant disorders, such as asthma which is a chronic, complex respiratory disease caused by various airway obstructions, airway eosinophil inflammation, and bronchial hyperresponsiveness [9].

Lactic acid bacteria (LAB), either live or heat-killed, have been reported to alleviate allergic symptoms by modulating Th1/Th2 responses toward a Th1-dominant state. Live *Lactobacillus paracasei* KW3110 administered orally to allergic mice revealed anti-allergic effects on both Th1 and Th2 cytokines, including IL-12 induction and IL-4 repression [10]. Heat-killed *Lactobacillus casei* strain Shirota (LcS) stimulated IL-12 secretion, which shifted the cytokine production pattern from a Th2 to a Th1 predominance and thereby suppressed IgE production [11], IgG1 responses, and systemic anaphylaxis [12] in human. Heat-killed *Lactobacillus brevis* SBC8803 inhibited IgE production and histamine secretion due to the improvement of the Th1/Th2 balance toward a Th1 dominance [13]. Heat-treated *Lactobacillus acidophilus* strain L-55 orally administered to ovalbumin (OVA)-sensitized BALB/c mice inhibited the nasal symptoms, sneezing and nasal rubbing, induced by OVA challenge [14]. Thus, either live or heat-killed LAB exhibited the capacity to ameliorate allergic responses in murine or in human.

The current study was aim to investigated the anti-allergy potential of *Lactobacillus plantarum* K37 (K37; DSM 27445) which is isolated from *fu-tsai*, a fermented food in Taiwan [15]. K37 was selected because of its profound immunomodulatory potency *in* *vitro* by inducing higher level of IFN-γ production in human peripheral blood mononuclear cells (hPBMCs) (unpublished data). Different amounts of heat-inactivated K37, 10^5^, 10^7^, and 10^9^ CFU, were orally administered to OVA-sensitized and OVA-challenged BALB/c mice. The effects of K37 on systemic allergy were investigated by measuring serum levels of Igs and cytokines. The AHR against methacholine were evaluated using non-invasive wholebody plethysmography. The histological analysis was also assessed.

## Materials and Methods

### Chemicals and Reagents

de Man, Rogosa, and Sharpe (MRS) broth was purchased from Difco (Spaarks, MD, USA). Methacholine, and OVA were purchased from Sigma-Aldrich (St. Louis, MO, USA). RPMI-1640 culture medium, fetal bovine serum (FBS), L-glutamate, antibiotics (penicillin, streptomycin, and amphotericin B) were obtained from (Gibco BRL, NY, USA). All other chemicals were purchased from Merck (Darmstadt, Germany).

### Preparation of *L. plantarum* K37

K37 was isolated from and deposited at DSME-Deutsche Sammlung Von Mikroorganismen Und Zellkulturen GmbH under accession number DSM 27445 [15]. K37 was grown in MRS broth at 30°C for 21 h and then harvested by washing and resuspending twice with sterile phosphate buffer saline (PBS). For heat-killed treatment, K37 was adjusted to 10^1^°CFU/mL in PBS, heat-treated at 100°C for 25 min using dry bath incubator (Evernew; Yu-Shing Biotech., Ltd, Taipei, Taiwan), and stored at −20°C until use.

### Ethics Statement

The animal experiments were approved by The National Yang-Ming University Institutional Animal Care and Use Committee (No. 1021276), and were carried out in strict accordance with the National Research Council’s Guide for the Care and Use of Laboratory Animals. All experimental procedures were performed under proper anesthesia and all efforts were made to minimize suffering of animals.

### Experimental Animals and Feeds

The OVA-sensitized and -challenged BALB/c mouse airway allergy model was performed in the current study. Four-week-old female BALB/c mice were purchased from the National Laboratory Animal Center, Taiwan, and maintained in National Yang-Ming University. The animals were housed in a temperature- and humidity-controlled room (at 25±2°C) with a 12-h light/dark cycle, with free access to a standard mouse/rat chow (LabDiet Autoclavable Rodent Diet 5010, PMI Nutrition International, Brentwood, USA) and water to acclimatize them for two weeks prior to OVA sensitization and K37 feeding.

**Figure 1:**
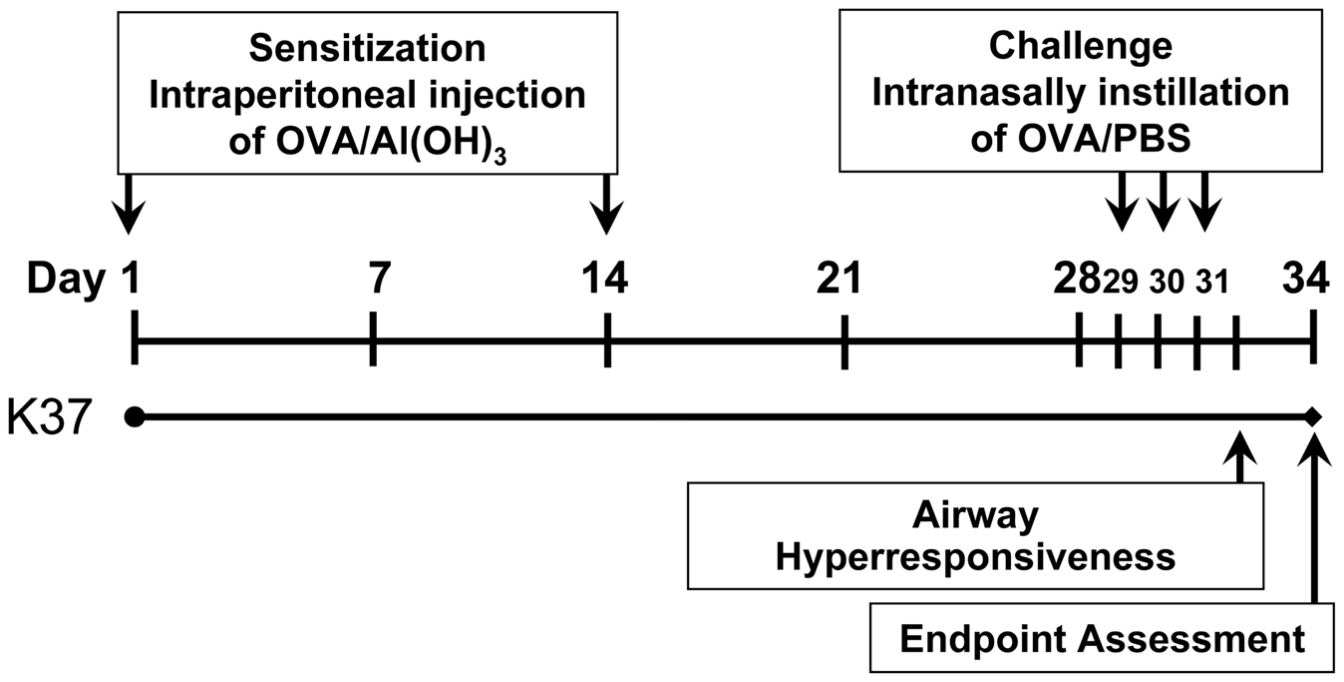
Experimental timeline of the ovalbimin (OVA)-sensitized BALB/c mouse model. Six-week-old female BALB/c mice were fed with heat-killed *Lactobacillus plantarum* K37 (K37) for 4 weeks and intraperitoneally injected three times at days 1 and 14 with 50 µg of OVA in 100 µL of Al(OH)_3_. On days 28, 29 and 30, the mice were challenged with OVA (1% in PBS, 100 µL/mouse) or PBS by intranasal administration. Serum was collected weekly for immunoglobulin (Ig) measurement. On day 34, mice were sacrificed and spleens were removed for spleen cell preparation.

To evaluate the anti-allergy effect of K37, the 6-week-old mice were sensitized and challenged with OVA to establish an OVA-induced airway allergy BALB/c mice model according to a published report [16] with slight modifications. The experimental procedure for administration of K37, OVA immunization, and sample collection is summarized in Figure 1. Five groups (n = 8 in each group) of mice were assigned a different treatment for 34 days. The healthy control (CON group) and allergy control (OVA group) groups were orally administered PBS (100 µL/mouse, day) using stainless gavaging tube. The other experimental groups (K37-L, K37-M, and K37-H) were orally administered by gavage with three doses of K37 in 100 µL PBS (10^5^, 10^7^, and 10^9^ CFU/mouse, day, respectively). All groups except for the healthy control group were intraperitoneally injected with 100 µL of aluminum hydroxide (Al(OH)_3_) containing 50 µg of OVA on days 1 and 14. The healthy control mice received Al(OH)_3_ only. On days 28, 29 and30, the mice were challenged with OVA (1% in PBS, 100 µL/mouse) or PBS by intranasal administration. On day 32, the AHR of the mice was measured. At the endpoint of assessment, all mice were sacrificed for bronchoalveolar lavage study. The spleen was removed in sterile condition for further culture. The lung was removed for histological analysis.

During the study period, the body weight of mouse was measured every day. There were no significant differences in food intake, feed efficiency, or changes in body weight among the groups. Blood was collected using retro-orbital venous plexus puncture and serum was prepared by centrifugation (3,000 *g*, 4°C, 10 min) on the designated day (Figure 1). The volume of sampling blood was less than 7.5% (v/v) of the total circulating blood volume of mouse which is in accordance with the Guidelines for Survival Bleeding of Mice and Rats for weekly blood collection. The serum was stored at −20°C until immunoglobulin analysis.

### Measurement of Airway Hyperresponsivenes (AHR)

The measurement of AHR was performed by wholebody plethysmography (Buxco, Troy, NY, USA) according to a previous report [17]. Pressure differences were measured between the main chamber of the plethysmograph, containing the animal and a reference chamber (box pressure signal). Mice were challenged with aerosolized normal saline (for the baseline measurement) or methacholine (6.25, 12.5, 25, and 50 mg/mL) for three minutes and readings were taken and averaged for three minutes after nebulization. The enhanced pause (Penh) ratio for each minute was recorded and after the third recorded value, the average Penh value was divided by the Penh of normal saline and was presented as a relative percentage increase of Penh.

### Analysis of Cell Population of BALF

At the endpint of assessment, the mice were sacrificed by using CO_2_ inhalation and the lungs were lavaged immediately via the trachea three times with 1 mL of Hanks’ balanced salt solution (HBSS). The BALF was cooled on ice and centrifuged (400 *g*, 4°C, 10 min). The supernatants were collected for the cytokine assay, and cell pellets were resuspended with 1 mL HBSS. The total numbers of cells in the BALF were counted with a standard haemocytometer. Cell counts of macrophage, eosinophil, neutrophil, and lymphocyte were performed by counting at least 200 cells in the cytocentrifuged preparations stained with Liu’s stain solution (Chi I Pao, Taipei, Taiwan), and differentiated by standard morphological criteria [18].

### Preparation of Spleen Cells

Spleen cell preparation was performed according to our previous report [19]. Briefly, after lung lavage, the spleens were removed aseptically from BALB/c mice and immediately immersed in RPMI 1640 medium supplemented with 100 IU/mL penicillin, 0.1 mg/mL streptomycin, and 0.25 µg/mL amphotericin B. The spleen was ground to be cells suspension with the above mentioned medium by using the flat buttom of syringe piston on 70 µm-cell strainer (Becton, Dickinson and Company; BD Biosciences, San Jose, CA, USA). The red blood cells were lysed with FACS lysing solution (Becton, Dickinson and Company; BD Biosciences, San Jose, CA, USA) and washed with HBSS for three times. After then, the spleen cells were adjusted to 1×10^6^ cells/mL in RPMI 1640 culture medium supplemented with 10% FBS, 1% L-glutamate, 100 IU/mL penicillin, 0.1 mg/mL streptomycin, and 0.25 µg/mL amphotericin B. The cells were cultured with phytohematoglutinin in a humidified incubator at 37°C with 5% CO_2_ for 48 h (Methods S1). After incubation, the supernatants were collected and stored at −20°C for further cytokine analysis.

### Measurement of Immunoglobulins and Cytokines by Enzyme-linked Immunosorbent Assay (ELISA)

The levels of total IgE and OVA-specific Igs (i.e. IgE, IgG1 and IgG2) were measured using the commercial ELISA kits (Bethyl Laboratory Inc., Montgomery, TX, USA, for total IgE and Alpha Diagnostic International Inc., San Antonio, TX, USA, for OVA-specific Igs) [20]. The concentrations of IL-2, IL-4, IL-5, IL-6, IL-12, IL-13, TNF-α, and IFN-γ were determined using ELISA procedure according to the manufacturers’ instructions (for IL-2, IL-4, IL-10, TNF-α and IFN-γ, eBioscience, Boston, MA, USA; IL-5, IL-6, IL-13 and eotaxin, R&D Systems, Minneapolis, MN, USA) [19].

### Histological Examination of Murine Lung Tissue

After lavage, the lungs were immediately removed and fixed in 10%(v/v) buffered formalin (in PBS, pH 7.4) for 24 h, and then embedded in paraffin. The fixed and embedded tissue was then stained with hematoxylin and eosin (H&E; Sigma, St. Louis, MO, USA) for histological assessment using light microscope (Leica DM750; Leica Microsystems, Heerbrugg, Switzerland).

### Statistical Analysis

Data were expressed as means ± the standard deviation (SD). The differences between means were tested for statistical significance using a one-way ANOVA followed by a Tukey’s post-hoc test. Differences between the OVA group and other groups were considered statistically significant when *P*<0.05 (*) or <0.01 (**).

## Results

### Effect of Oral Administration of K37 on Immunoglobulin Production in OVA-Sensitized Mice

**Figure 2:**
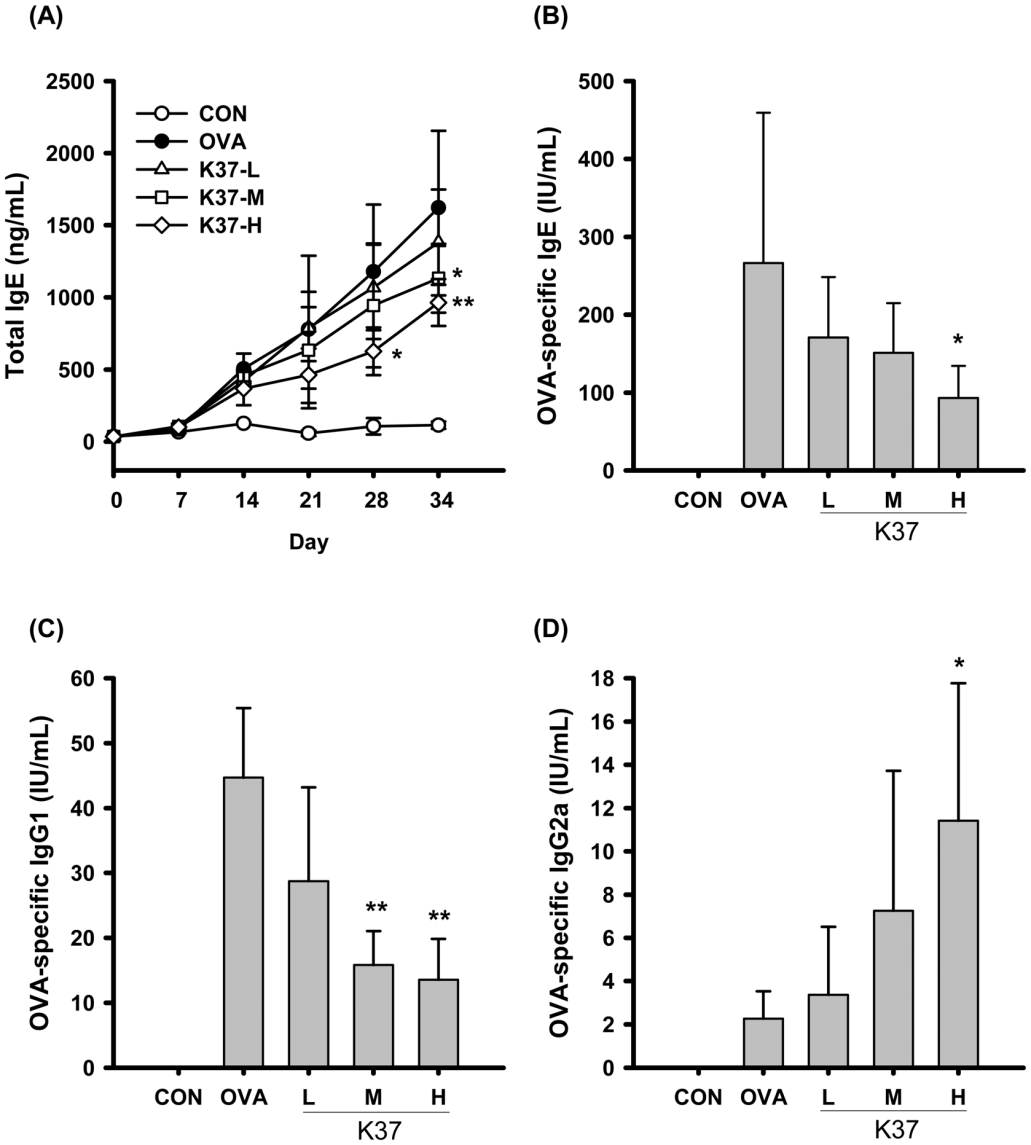
Effect of oral administration of K37 on Ig production in OVA-sensitized mouse serum. Serum levels of (A) total IgE, (B) OVA-specific IgE, (C) OVA-specific IgG1, and (D) OVA-specific IgG2a were determined by ELISA. Each value represents the mean ± SD, (n = 8). A difference between K37 groups and OVA group was considered statistically significant when *P<*0.05 (*) and *P<*0.01 (**).

The levels of serum immunoglobulins were first investigated to shed light on the effects of LAB on OVA-sensitized mice. In the present study, mice were orally administered with 10^5^, 10^7^, and 10^9^ CFU/day (K37-L, K37-M, and K37-H, respectively) for 34 days and intraperitoneally injected with OVA/Al(OH)_3_ on days 1 and 14 (Figure 1). As shown in Figure 2A, the total serum IgE in OVA-sensitized mice elevated after day 7 and continued to increase through day 34. K37-M and K37-H groups both showed significantly reduced IgE levels on day 34 when compared with OVA group (Figure 2A, *P*<0.05 and 0.01, respectively). The K37-H showed prominent effect on lowering serum level of OVA-specific IgE (Figure 2B) on day 34 compared with the OVA-sensitized group (OVA) (*P*<0.01). The serum level of OVA-specific IgG1, the Th2-type immunoglobulin, in both K37-M and K37-H groups were markedly lower than that in the OVA group by about 3 folds (Figure 2C; *P*<0.01). K37 groups had increased serum levels of OVA-specific IgG2a, the Th1-type immunoglobulin. When compared with that in the OVA group, the level of OVA-specific IgG2a in the K37-H group showed statistically significant difference (*P*<0.05; Figure 2D).

### Airway Hyperresponsiveness

**Figure 3:**
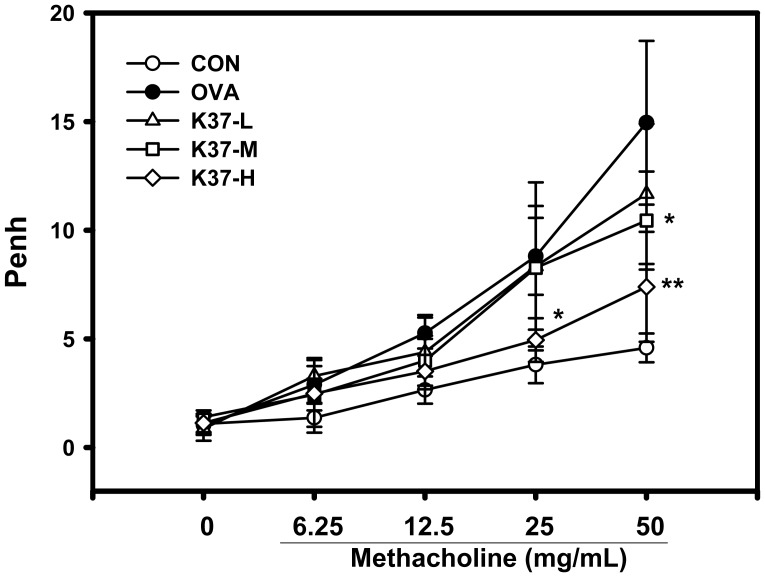
Effect of oral administration of K37 on the airway response to aerosolized methacholine measured 24 h after the last OVA challenge as expressed by Penh. A difference between K37 groups and OVA group was considered statistically significant when *P<*0.05 (*) and *P<*0.01 (**).

To evaluate the effects of K37 on AHR, the assessment was performed using non-invasive wholebody plethysmography 1 day after the final challenge. As shown in Figure 3, BALB/c mice sensitized intraperitoneally and challenged intranasally with OVA revealed increases in the Penh value (OVA and K37 groups) in response to methacholine inhalation compared with PBS-sensitized and PBS-challenged mice (CON group). However, oral administration of K37 alleviated the development of AHR compared with the OVA group. The Penh levels of the K37-H group were similar to those of the CON group and significantly lower than those in the OVA group (methacholine 25 mg/mL, *P*<0.05; 50 mg/mL, *P*<0.01). The K37-M group showed a significantly lower Penh level at 50 mg/ml methacholine (*P*<0.05).

### Cell Population of BALF

**Figure 4:**
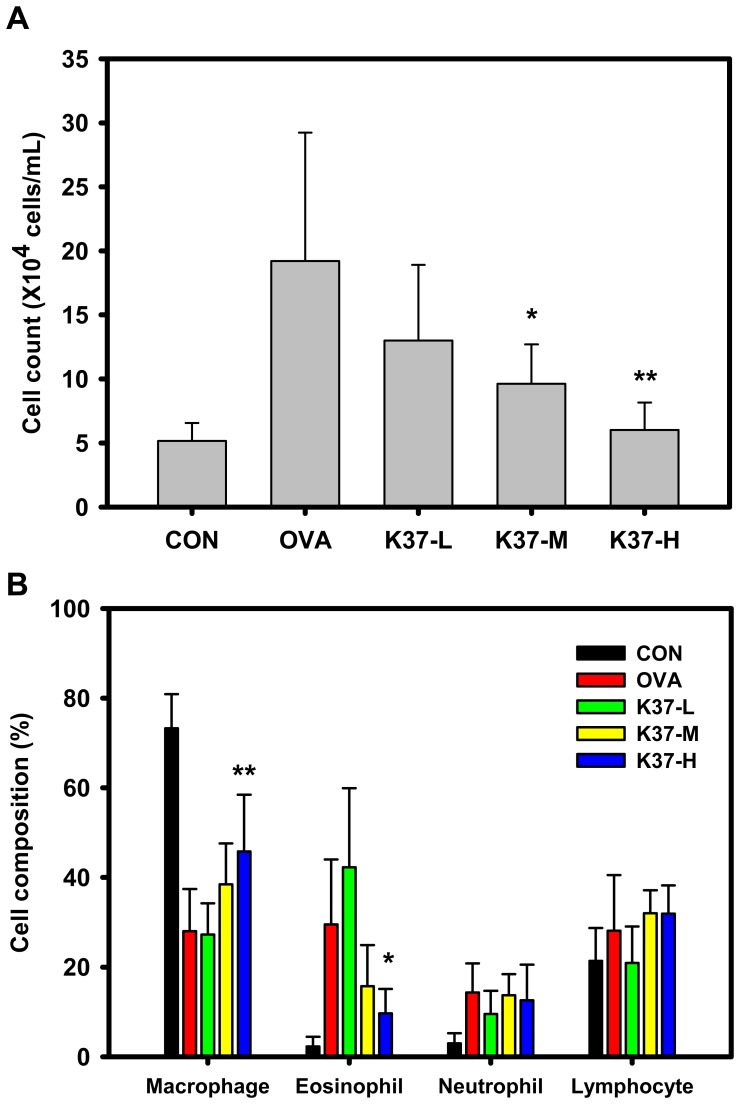
Effect of oral administration of K37 on lung tissue inflammatory cell infiltration in OVA-sensitized mice after OVA challenge. (A) Total counts of cells in BALF from healthy control (CON), allergy control (OVA), and K37 (K37-L, K37-M, and K37-H) groups of mice. (B) Cell population of macrophage, eosinophil, neutrophil and lymphocyte in BALF were analysed and are expressed as the mean ± SD, (n = 8). A difference between K37 groups and OVA group was considered statistically significant when *P<*0.05 (*) and *P<*0.01 (**).

The numbers of macrophages, eosinophils, neutrophils and lymphocytes were counted to obtain the cell population of BALF for evaluating the effects of K37 on lung inflamamtion. The influx of inflammatory cells into lungs was examined. As shown in Figure 4A, the number of cells in both K37-M and K37-H groups were significantly lower when compared with that in the OVA group (*P*<0.05). The cell population of BALF was further analyzed (Figure 4B). In the OVA group, the percentage of eosinophils and neutrophils were significantly increased while the percentage of macrohages was decreased compared with the CON group. As for the K37-H group, the population of macrophage is elevated (*P*<0.01) while eosinophil infiltration is lowered (*P*<0.05) when compared with the OVA group.

### Histologic Examination of Murine Lung Tissue

**Figure 5:**
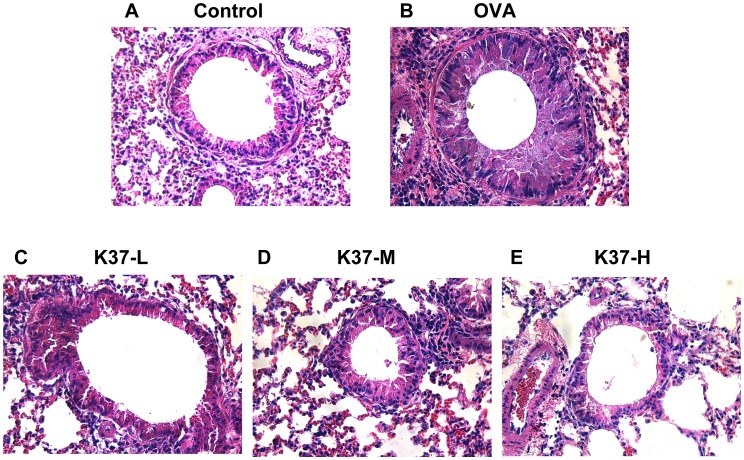
Effect of oral administration of K37 on lung tissue inflammatory cell infiltration and airway remodeling in OVA-sensitized mice after OVA challenge. Representative hematoxylin and eosin (H&E) stained section of lung tissue experimental mice (magnification 40×).

The effect of K37 on lung inflammation in OVA-sensitized and OVA-challenged mice was further evaluated with histological examination. As shown in Figure 5, upon H&E staining, inflammatory changes such as increase in cell infiltration and thickness of epithelial cells were observed in OVA group (Figure 5B). The inflammation in the peribronchial and perivascular regions of mice orally administered with K37 was significantly improved compared with that in the OVA group because of fewer cell infiltration and thiner epithelial layer (Figure 5C–E).

### Effects of Oral Administration of K37 on Cytokine Levels in BALF and Spleen Cell Culture from OVA-sensitized Mice

**Figure 6:**
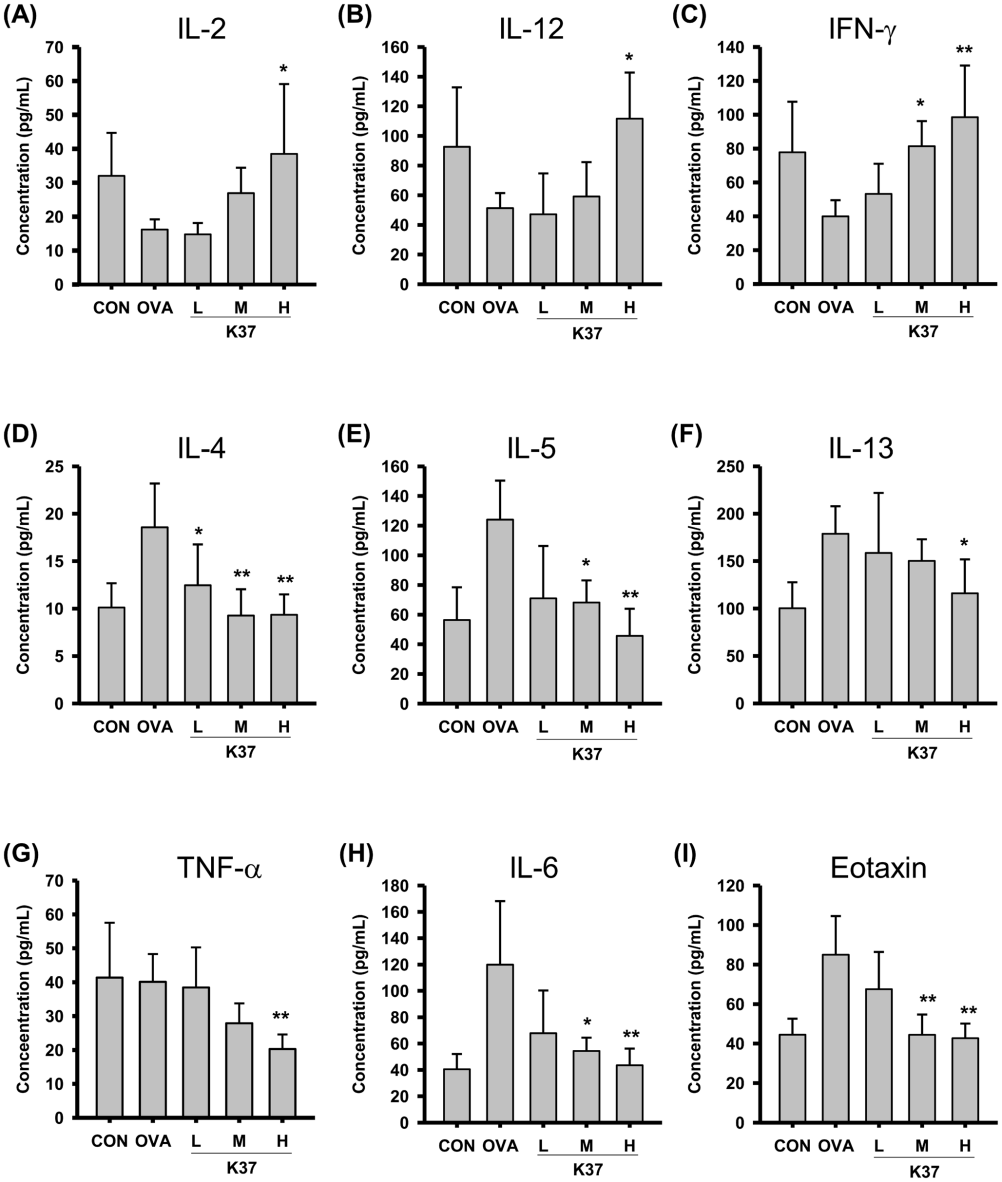
Effect of oral administration of K37 on cytokines production in BALF of OVA-sensitized mice. The concentration of (A) IL-2, (B) IL-12, (C) IFN-γ, (D) IL-4, (E) IL-5, (F) IL-13, (G) TNF-α, (H) IL-6, and (I) eotaxin in the BALF were determined by ELISA. Each value represents the mean ± SD, n = 8. A difference between K37 groups and OVA group was considered statistically significant when *P<*0.05 (*) and *P<*0.01 (**).

**Figure 7:**
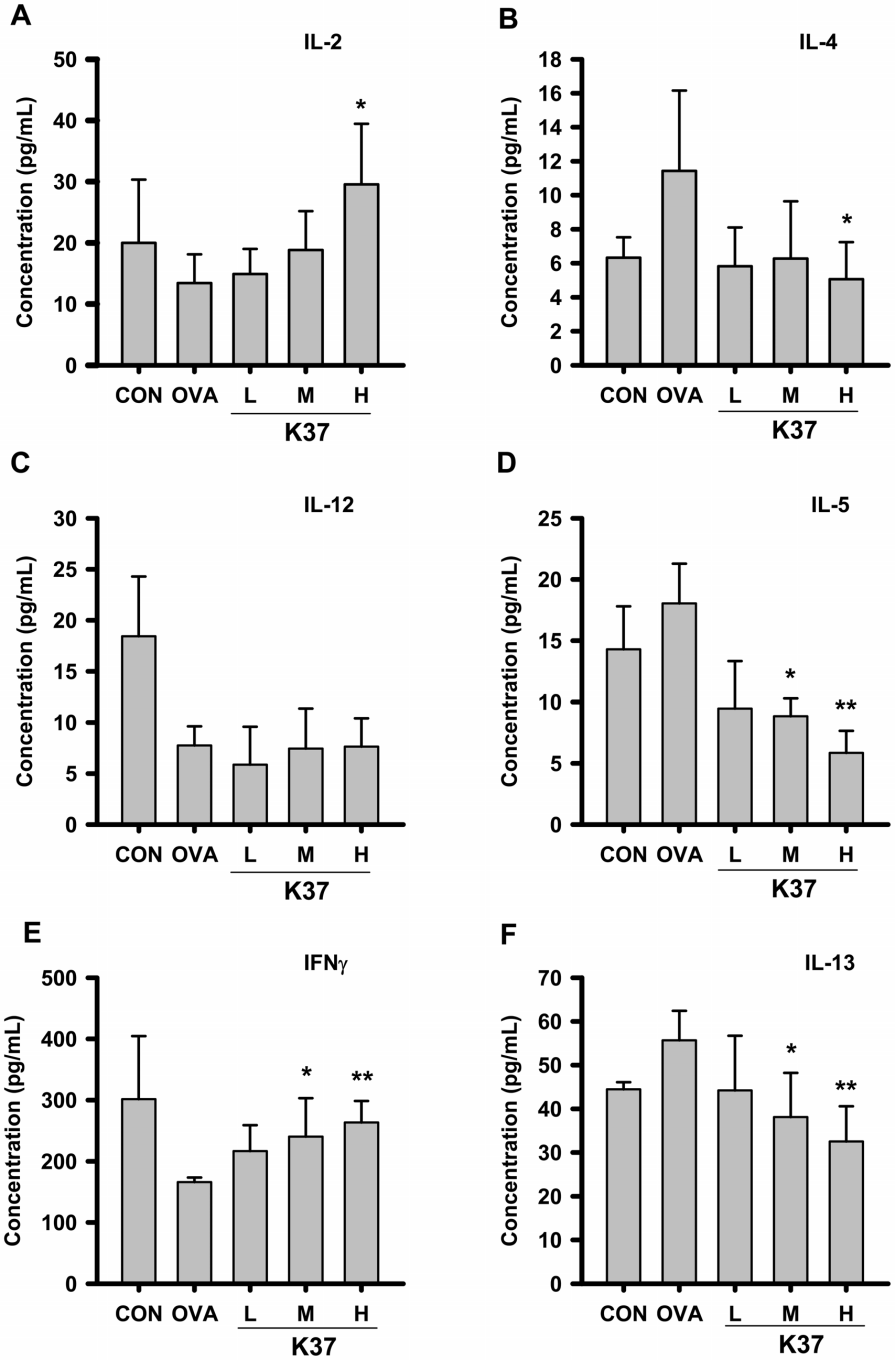
Effect of oral administration of K37 on cytokines production in spleen cells of OVA-sensitized mice. The concentration of (A) IL-2, (B) IL-4, (C) IL-12, (D) IL-5, (E) IFN-γ, and (F) IL-13 in the BALF were determined by ELISA. Each value represents the mean ± SD, n = 8. A difference between K37 groups and OVA group was considered statistically significant when *P<*0.05 (*) and *P<*0.01 (**).

The cytokine production profile was employed to evaluate the effects of K37 on T-cell responses. The concentrations of Th1 cytokines, IL-2, IL-12, and IFN-γ, and Th2 cytokines, IL-4, IL-5, and IL-13, in BALF (Figure 6) and in spleen cell cultures (Figure 7) were measured using the ELISA method. As shown in Figure 6A–C, levels of Th1 cytokines, IL-2, IL-12, and IFN-γ, in BALF were elevated dose-dependently in K37 groups compared with the OVA group. In the K37-H group, the levels of IL-2 and IL-12 were significantly higher than those in the OVA group (Figure 6A and 6C, *P*<0.05). The IFN-γ levels were significantly increased in both K37-M (*P*<0.05) and K37-H groups (*P*<0.01). Levels of Th2 cytokines in BALF, including IL-4, IL-5, and IL-13, were also measured. The levels of IL-4 in BALF of K37 groups (Figure 6D) were significantly decreased compared with that in the OVA group (K37-L, *P*<0.05; K37-M and K37-H, *P*<0.01). In both K37-M and K37-H groups, the levels of IL-5 were significantly lower than that in the OVA group (Figure 6C; K37-M, *P*<0.05 and K37-H, *P*<0.01). However, only in the K37-H group was the level of IL-13 significantly diminished (Figure 6F).

As for the cytokines in spleen cells, the levels of IL-2 (Figure 7A) in K37 groups were elevated compared with that in the OVA group; however, only the increase in the K37-H group showed statistical significance (*P*<0.05). In all OVA-sensitized groups (OVA and K37 groups), the levels of IL-12 were comparable and lower than that in the non-sensitized group (CON group) (Figure 7C). In both K37-M and K37-H groups, the levels of IFN-γ were higher than that in the OVA group, showing statistical significance (*P*<0.05 and *P*<0.01, respectively; Figure 7E). The reduced IL-4 levels were observed in K37 groups compared with OVA group (K37-H, *P*<0.05). The levels of IL-5 and IL-13 were decreased dose-dependently in K37 groups compared with OVA group (*P*<0.05 for K37-M and *P*<0.01 for K37-H; Figure 7D and 7F). Taken together, the results of cytokine determination suggested that K37 treatment induced the production of Th1 cytokines, IL-2 and IFN-γ, in BALF and spleen cell culture. Diminished secretion of Th2 cytokines, IL-4, IL-5 and IL-13, was observed in K37 groups of both BALF and spleen cell culture.

The levels of inflammatory mediators, TNF-α, IL-6 and eotaxin, in BALF were also measured to evaluate the effects of K37 on inflammation status in lung. As shown in Figure 6G, the level of TNF-α in BALF of each group was comparable, only in K37-H, it was significantly lower than OVA group (*P*<0.01). The levels of IL-6 and eotaxin were dramatically elevated in the OVA group compared with the CON group (Figure 6H and 6I, repectively). However, the levels of IL-6 and eotaxin in K37-H group were comparable to those in CON groups. The levels of TNF-α and IL-6 in spleen cell culture showed the same tendency as in BALF (data not shown). Taken together, K37 treatment decreased the production of TNF-α, IL-6 and eotaxin in OVA-sensitized mice.

## Discussion

This study investigated the effects of orally administered heat-inactivated K37 on OVA-induced allergic asthma in BALB/c mice model. The present results showed that K37 suppressed allergic parameters, including AHR, airway inflammation, total IgE and OVA-specific IgE. The cytokine production profile in BALF and spleen cell culture revealed that K37 skewed immune responses toward Th1 responses, elevated levels of Th1 cytokines, and diminished levels of Th2 cytokines. The levels of inflammatory mediators in BALF, TNF-α, IL-6 and eotaxins, were dose-dependently decreased by K37 oral administration. The lower numbers of eosinophils and neutrophils in BALF suggested that inflammation was ameliorated by K37. The histological observation on lung sections also showed infiltration of fewer cells.

Ovalbumin is the most frequently used allergen in animal models of experimental allergy and the elevated levels of IgE and OVA-specific Igs in serum were observed in OVA-sensitized animals. In the current study, the intraperitoneally OVA-sensitized and intranasally OVA-challenged BALB/c model (Figure 1) was employed to investigate the anti-allergic effects of K37. As shown in Figure 2, the increasing levels of serum IgE, OVA-specific IgE, and OVA-specific IgG1 in the OVA group indicated the allergy animal model was established and represented B-cell type Th2 responses. In K37 groups, the levels of total IgE, OVA-specific IgE, and OVA-specific IgG1 were significantly lower than in the OVA group at the endpoint of assessment (K37-M, *P*<0.05; K37-H, *P*<0.01) (Figure 2). Furthermore, considerable increase of OVA-specific IgG2a was observed in the K37 group (Figure 2D). The results of serum Igs analysis demonstrated systemic anti-allergic effects of K37. Moreover, the modulatory effects of K37 on OVA-induced immunoglobulins secretion showed a dose-dependence.

Some LAB strains with Th1-dominant responses were reported to be effective in modulating the production of OVA-induced immune responses [2], [20]. Our previous report showed that *Lactococcus lactis* A17 with Th1-polarizing potential, both alive and heat-killed, could ameliorate systemic allergy symptoms in OVA-sensitized BALB/c mice [20]. Consequently, it was speculated that heat-killed LAB with an *in* *vitro* Th1-polarizing potential might exhibit anti-allergic effects *in* *vivo*. In current study, K37 which was previously evaluated for the effect on *in* *vitro* cytokine production in hPBMCs and showed greater IFN-γ production in hPBMCs than *L. lactis* A17 (data not shown) was investigated for anti-allergy and anti-asthma activities.

The relevance of LAB to human health has gained worldwide attention. There are increasing evidences on biological activities of LAB reported, including anti-influenza virus infection [21], anti-allergy [10], anti-cancer [22], and anti-inflammation [19]. As for anti-allergy activity, different species of LAB may exhibit different responses. Hougee et al. (2010) investigated the anti-allergic effects of *Bifidobacterium breve* M-16V and *Lactobacillus plantarum* NumRes8 and found that both bacteria reduced the numbers of eosinophils and lowered the levels of OVA-specific IgE in OVA-sensitized mice. However, only *B. breve* M-16V can reduce the secretion of IL-4 and IL-5, both being Th-2 cytokines [23]. Except the species dependence, the beneficial effects of LAB may also be strains depedent. In the current study, K37 was investigated the potential for anti-allergy. Some strains of *L. plantarum* were investigated their anti-allergy effects. Oral administration of *L. plantarum* NRIC0380 lyophilized powder was reported to suppress IgE production in β-lactoglobulin-immunized BALB/c mice [24] and the induction of regulatory T cell (CD4^+^CD25^+^Foxp3^+^; Treg) was involved in the anti-allergy activity [25]. The lyophilized *L. plantarum* CJLP 133 was reported to exhibit therapeutic potential to treat house-dust mite-induced dermatitis in NC/Nga mice through Th-1 and Treg activation [26]. In addition to live or lyophilized *L. plantarum*, heat-killed *L. plantarum* was also investigated the anti-allergy activity. Heat-killed *L. plantarum* KTCT 3104, when orally administered with a daily dose of 5×10^7^ CFU/mouse, was reported to reduce OVA-induced AHR by the reduction of Th-2 cytokines, IL-4 amd IL-5, and an enhancement of Th-1 cytokine, IFN-γ. As for the levels of Igs in serum, *L. plantarum* KTCT 3104 decreased the OVA-specific IgE. However, OVA-specific IgG2a which was a Th1 type B cell response was also decreased [27]. In the current study, the levels of IgE was significantly lower in K37-M and K37-H groups (Figure 2A) which represented the systemic allergy was reduced. The *in* *vivo* Th1-polarizing effects of K37 were evidenced by increased levels of OVA-specific IgG2a and Th1 cytokines, IL-2 and IFN-γ, both in BALF and spleen cells culture. The current results revealed that heat-killed K37 exhibited both systemic and airway anti-allergic effects via Th1-polarizing effects and K37-H showed the best activity, suggesting that the anti-allergic effects of K37 was dose-dependent.

LAB either live or heat-killed has been reported to be effectively useful in experimental allergy animal models. Our previous report showed that both live and heat-killed *L. lactis* A17, isolated from Taiwan fermented cabbage, exhibited systemic anti-allergic effects in OVA-sensitized mice [20]. Orally administered heat-killed *Lactobacillus pentosus* strain S-PT84, which is a IL-12-inducing LAB, lowered serum IgE levels and splenic IL-4 production in OVA-induced allergy BALB/c mice [28]. Inhibition on IgE and histamine was observed for heat-killed *L. brevis* SBC8803 in improvement of the Th1/Th2 balance toward Th1 dominance [13]. However, it is believed that the use of non-viable LAB as biological response modifiers exhibits several attractive advantages; such products would be safe and have a long shelf-life [29]. In the current study, we used heat-killed K37 because it was easy to storage and will have a longer shelf-life than viable LAB. As for heat-killed treatment, some heat-resistant components, such as intracellular or cell wall components of LAB, resposible for the biological activities are existed. However, further investigations are needed for identifying the active components.

Hyperresponsiveness is defined as increased sensitivity to some cholinergic agents, like methacholine, which leads to smooth muscle constrictions and increases airway resistance by narrowing the airways [30], [31]. Asthma is one of the airway hyperresponsive diseases which can be characterized by the accumulation of inflammatory cells, increase in mucus production, release of certain Th2 cytokines, IL-4, IL-5, and IL-13, and increased levels of IgE [27]. In the current study, the changes in airway remodeling of K37 treatment were investigated. K37 markedly alleviated the OVA-induced AHR to inhaled methacholine (Figure 3). According to lung histopathological studies using H&E staining, inflammatory cell infiltration was inhibited in the K37 groups compared with the OVA group (Figure 5). Many types of inflammatory cells are involved in the process of airway inflammation, such as mast cells, eosinophils, and T lymphocytes [32]. Among those cells, eosinophils play the crucial role in the pathogenesis of allergic diseases. Eosinophils are attracted via CC chemokine receptor 3 (CCR3) to chemoattractants, such as eotaxin released in the airway [33]. Clinical and experimental studies have established eosinophilia as a sign of allergic disorders [34]. In our result, the number of eosinophils was significantly decreased in the K37-H group compared with the OVA group (Figure 4B). The infiltration of eosinophils was observed in the peribronchial regions of the lung section of the OVA group. However, fewer cells infiltrated in K37 groups (Figure 5). Moreover, eotaxin is also regarded as an important aspect of allergy because it induces the recruitment of eosinophils, basophils, and Th2 lymphocytes in lungs [35 36]. The levels of eotaxin in BALF of K37-M and K37-H groups are comparable to that in the CON group (Figure 6I). As for the study of LAB on allergic infiltrated eosinophils, *Bifidobacterium animalis* was reported to have moderately reduced effects on the number of infiltrating eosinophils and lymphocytes in the lungs; however, *B. animalis* had no effects on allergen-specific serum IgE levels [36]. However, K37 not only reduced the number of infiltrating eisonophils but also lowered the levels of OVA-specific IgE (Figure 2B) as well as total IgE (Figure 2A).

Cytokine expression, such as IL-4, IL-5, and IL-13, in connection with T-cell response, and IgG1 production in connection with B-cell response [37], are thought to be related to Th2 immunity. Th2 cytokines play important roles in asthma. Among Th-2 cytokines, IL-4 drives naïve Th cells to be the Th2 phenotype and induces B cells to switch the isotype to IgE. IL-5 produced by Th2 cells is responsible for eosinophil growth, differentiation, mobilization, recruitment, activation and survival [6]. IL-13 plays a critical role in the pathogenesis of asthma [38]. Excessive production of IL-4, IL-5, and IL-13 was implicated in the development of asthma [39]. The current results showed increased T-cell responsive Th2 cytokines, IL-4, IL-5 and IL-13, in both BALF and cultured spleen cells from OVA groups (Figure 5 and Figure 6, respectively). Evidences have shown that Th1/Th2 regulatory effects of LAB is useful in allergy animal model. Increase in production of Th1 cytokines, IL-12 and IFN-γ as well as decrease in Th2 cytokines, IL-4 and IL-5 by LcS [40] contributed to the prevention of seasonal allergy in Japan [41]. Another anti-allergic LAB, *L. paracasei* strain KW3110, was reported to inhibit the production of Th2 cytokines, IL-4, IL-5 and IL-13 [42]. In the present study, AHR was decreased by oral administration of K37, suggesting its anti-allergy effects which may be attributed to the reduction of Th2 cytokines and eotaxin well known to play important roles in the development of AHR [38]. It was speculated that the inhibitory effects on IL-13 contributed to the anti-allergic activity of K37 (Figures 6F and 7F). In addition to the inhibition on Th2 cytokines, K37 also enhanced production of Th1 cytokines. The production of IFN-γ production in hPBMCs was elevated by K37 (data not shown). K37 also increased the levels of IL-2 and IFN-γ in BALF and spleen cell culture (Figures 6 and 7, respectively). Taken together, the current results indicate that K37 has a promising effect on modulating T-cell responses in OVA-sensitized mice toward Th1 responses.

Increased levels of pro-inflammatory cytokines in BALF of OVA-sensitized animals were observed in reports, such as IL-1β, IL-6, IL-17 and TNF-α [43]–[46]. In the current study, we analyzed the levels of TNF-α and IL-6 in BALF to further evaluate the inflammation in lung. The levels of IL-6 were reduced in K37 groups compared with OVA group (Figure 6H). However, the level of TNF-α in BALF of each group was comparable, only in K37-H, it was significantly lower than OVA group (*P*<0.01) The detailed mechanism for the reduction of TNF-α in K37 groups still need further investigation. The recent results showed the decreased levels of TNF-α and IL-6 in BALF of the K37-H group which represented inflammation in lung was reduced (Figure 6). In K37 groups, fewer cell infiltration and thiner epithelial layer was observed in lung section (Figure 5). Taken together, these results suggested that the amelioration of inflammation in lung contributed to the anti-allergic effects of K37.

In summary, the current study demonstrated that K37 could ameliorate asthma-like responses in OVA-sensitized BALB/c mice. In the evaluation of AHR by means of enhanced pause using wholebody plethysmography, it was found that the administration of K37 could significantly decrease AHR in OVA-immunized BALB/c mice. K37 induced pronounced immunomodulatory effects on most of the parameters tested. We concluded K37 to be a promising candidate for protection from and prophylactic treatment of allergic diseases.

## Supporting Information

Methods S1 **Spleen cells culture procedure for cytokine analysis.**
(DOCX)
