# Supplementary material for: Oral Administration of Heat-Inactivated Lactobacillus plantarum K37 Modulated Airway Hyperresponsiveness in Ovalbumin-Sensitized BALB/c Mice
Source: PLoS One. 2014 Jun 17;9(6):e100105. doi: 10.1371/journal.pone.0100105 (PMC4061068; doi:10.1371/journal.pone.0100105)
Supplement: Methods S1 — Spleen cells culture procedure for cytokine analysis. (DOCX) [file pone.0100105.s001.docx]

**Methods S1. Spleen cells culture procedure for cytokine analysis**

Spleen cells were adjusted to 1 × 10^6^ cells/mL in RPMI 1640 culture medium supplemented with 10% FBS, 1% L-glutamate, 100 IU/mL penicillin, 0.1 mg/mL streptomycin, and 0.25 μg/mL amphotericin B. Cells were plated at a density of 5×10^5^ cells/well in 24-well plates and were incubated with 2 μg/mL phytohemagglutinin (PHA; 0.5 mL/well) in a humidified incubator at 37°C with 5% CO_2_ for 48 h. After incubation, the supernatants were collected and stored at -20°C for further cytokine analysis.
